# Cancer Pain: Radiotherapy as a Double-Edged Sword

**DOI:** 10.3390/ijms26115223

**Published:** 2025-05-29

**Authors:** Monika Konopka-Filippow, Barbara Politynska, Anna M. Wojtukiewicz, Marek Z. Wojtukiewicz

**Affiliations:** 1 Department of Oncology, Medical University of Bialystok, 15-089 Bialystok, Poland; monikafilippow@gmail.com; 2 Department of Psychology and Philosophy, Medical University of Bialystok,15-420 Bialystok, Poland; bpolitynska@wp.pl (B.P.); aniawojtukiewicz@gmail.com (A.M.W.); 3 Robinson College, University of Cambridge, Cambridge CB3 9AN, UK

**Keywords:** pain, neuropathic pain, radiotherapy, bone metastases

## Abstract

Cancer pain is a common issue for patients, especially in the advanced stages of cancer, and significantly affects the quality of life (QoL), treatment tolerance, and overall treatment outcomes. Pain may be caused by primary tumors, metastases, or as a consequence of the inflammatory reaction of tissues surrounding the tumor following radiotherapy (RT). Effective pain management is crucial, especially with RT being a key method for alleviating cancer pain, particularly in cases of bone and soft tissue metastases. RT provides relief for 60–80% of patients by reducing tumor size and mitigating associated pain. Radiotherapy itself can also induce pain, especially radiation-induced neuropathic pain, which may require further treatment. Despite these potential side effects, RT remains an essential tool in managing cancer pain, though careful management of its toxicities is necessary to improve patient QoL and survival.

## 1. Introduction

Most cancer patients experience pain as part of their illness, especially those with advanced cancer, where the goal of treatment is not complete recovery, but providing relief from symptoms, including pain. Cancer pain may be the result of a developing primary tumor or metastases in the lymph nodes, but predominantly it occurs in patients experiencing distant metastases, particularly in the skeletal system [1]. It has long been established that pain has a negative impact on quality of life (QoL), decreasing the tolerance of treatment and can also affect treatment compliance [2]. The latter substantially influences the treatment results and patient outcomes [3]. Moreover, cancer pain may appear during or after oncological therapy, including radiotherapy (RT). Therefore, appropriate pain management plays an important role in the overall outcome of anticancer therapy with regard to patient treatment tolerance and their QoL [4].

## 2. Radiotherapy as a Mainstream Procedure for the Treatment of Cancer Pain

The main RT techniques used for the management of cancer pain are as follows:

1. Teleradiotherapy (or external beam radiation therapy, EBRT) is the most commonly used irradiation technique in oncological treatment. In EBRT, the radiation source is placed at a certain distance from the tumor, and the patient, lying in a predetermined position, is exposed to a beam of ionizing radiation (either photon or electron) generated by a therapeutic device—a medical linear accelerator. The irradiation area includes not only the tumor itself, but also an appropriate margin of surrounding tissue, which has been visualized in imaging studies (computed tomography and magnetic resonance imaging), in order to account for the possibility that cancerous cells may have also occupied areas in the vicinity of the tumor. A patient who is to undergo EBRT is prepared for the procedure by means of selecting the appropriate form of immobilization and conducting a computed tomography-guided localization scan to define the exact irradiation targets. Additionally, important points are marked on the patient’s skin, which are essential for reproducing the planned irradiation field [5]. The basic EBRT technique is 3D conformal radiation therapy (3D-CRT), which uses fixed angles to deliver radiation in discrete steps from multiple directions, with the beam shapes and angles adjusted to match the tumor’s three-dimensional structure. Here, 3D-CRT does not modulate the intensity of the radiation during treatment. Volumetric modulated arc therapy (VMAT) is a more sophisticated technique that delivers radiation in a continuous, arc-based rotation around the patient from the treatment source, often a linear accelerator (LINAC), which rotates 360 degrees around the patient while adjusting the intensity of the radiation beam as it moves. VMAT is a conformal radiotherapy technique, which means that the radiation beam and dose are more closely adapted to the shape and size of the tumor than in 3D-CRT. Traditional RT techniques, primarily based on photon (X-ray) therapy, often affect healthy tissues adjacent to the tumor, leading to unintended side effects. More recently, image-guided RT (IGRT) and stereotactic radiosurgery (SRS) have made strides in real-time tumor tracking and high-precision delivery, enabling the treatment of tumors in challenging locations with sub-millimeter accuracy.
2. Brachytherapy (BRT) is a form of localized RT used to treat various types of cancer, which involves placing a radioactive source directly inside or very close to the tumor. This allows for high doses of radiation to be delivered directly to the cancerous tissue, simultaneously minimizing radiation exposure to surrounding healthy tissues. The localized radiation can help shrink tumors, alleviate obstruction, and reduce inflammation, which may lead to significant pain relief [6]. This technique is particularly effective in cases where cancer causes localized pain due to soft tissue masses; for instance, in head and neck cancers, prostate cancers, and gynecological malignancies [7]. Sometimes BRT is used to treat painful bone metastases, particularly in areas like the spine, pelvis, or other bones where radiation can be delivered precisely [8]. In cancers of the gastrointestinal tract, urinary tract, or reproductive organs, BRT can be used to treat obstruction-related pain, especially when tumors exert pressure on other organs or nerves. Various radioactive isotopes are used in BRT, such as iridium-192 (192Ir), cesium-137 (137Cs), cobalt-60 (60Co), radium-226 (226Ra), or yttrium-90 (90Y), which are placed in close proximity to the tumor for a strictly defined period of time. The various types of BRT are described in Table 1.

Brachytherapy is considered an invasive radiotherapy technique and often requires collaboration with an anesthesiologist and a nursing assistant to monitor the appropriateness of anesthetic procedures. During brachytherapy, a relatively high dose of radiation is delivered to a small volume of tissue surrounding the radiation source applicators, and the treatment effects experienced by the patient typically occur within a few days [14,15]. The main limitations of BRT are that its use is restricted to managing localized pain and tumors that are accessible for radiation delivery, thus, BRT is rarely used as a treatment option for painful bone metastases.

3. Proton and heavy ion therapy are some of the most significant developments in RT. These novel therapies use charged particles—protons (positively charged particles—hydrogen nuclei) and heavier ions (such as carbon ions)—to deliver radiation to tumors. Compared to traditional photon therapy, proton and heavy ion therapy offer remarkable benefits, especially in terms of reducing radiation exposure to surrounding healthy tissues and minimizing side effects. Protons have mass and energy, and when they enter tissue, they deposit most of their energy at a specific depth known as the Bragg peak. This allows the proton beam to release a significant portion of its radiation directly at the tumor site, with little to no radiation affecting the tissues beyond the tumor. As a result, healthy organs and structures surrounding the tumor are spared from unnecessary radiation exposure. This precise dose distribution is particularly valuable when treating tumors located near critical structures such as the brain, spinal cord, eyes, and especially in pediatric cancers, where minimizing side effects is of utmost importance [16,17].
  Heavy ion therapy, often involving carbon ions, takes the precision of proton therapy a step further. These ions are heavier than protons and possess greater energy, allowing them to deliver a higher biological effectiveness (i.e., having higher cell-death potential) at the same physical dose. Carbon ions can penetrate deeper into tissue while maintaining their precise dose distribution, making them ideal for treating tumors in challenging locations, including those that are resistant to conventional radiation [18]. One of the distinct advantages of heavy ion therapy over proton therapy is its ability to overcome the resistance of hypoxic tumor cells—cells that are deprived of oxygen, which are often more resistant to conventional radiation [19]. These techniques, due to their high cost and limited availability, should be selected for use in patients with oligometastatic/oligorecurrent disease with a low metastatic burden [20,21]. To date, proton therapy has been used with encouraging results for the treatment of bone or liver metastases, as an option in which the reduced radiation exposure to normal tissues leads to a clinically significant reduction in treatment-related toxicities [22]. It is hoped that the increase in the number of proton therapy treatment centers available worldwide will potentially lead to an expansion of its commissioning to include indications that are currently not routinely funded [23].
  The future of RT appears promising, with continued advancements in technology that aim to further improve precision and reduce toxicity. The development of FLASH RT, which involves delivering very high doses of radiation at ultra-fast speeds (ultra-high dose rates ≥ 40 Gy/s), is one such breakthrough that is currently being explored. The preliminary data suggest that the lower levels of toxic oxygen reactive species in normal tissues may explain why fewer side effects may be produced by FLASH than by conventional RT [24]. The first-in-human FAST-01 clinical trial demonstrated the clinical feasibility of proton FLASH in the treatment of extremity bone metastases [25]. The FAST-02 trial is currently assessing the toxicities of treatment (eight Gy in a single fraction) and pain relief in patients with painful thoracic bone metastases [26]. FLASH therapy has shown potential for sparing normal tissues from radiation toxicity while still effectively targeting tumors [27].
  Furthermore, as proton and heavy ion therapy centers become more accessible and cost-effective, it is expected that more patients will benefit from these advanced treatments. In addition, personalized medicine—where treatment is tailored to the individual characteristics of both the patient and the tumor—will continue to play an essential role in RT.

Recently, even more targeted approaches have been introduced, such as radioisotope therapy, which directly destroys cancer cells adjacent to the nuclide. Its therapeutic action is based on the emission of beta radiation, which destroys cancer cells with minimal damage to the surrounding healthy tissues [28]. In radioisotope therapy, the most commonly used elements are strontium-89 (89Sr), samarium-153 (153Sm), phosphorus-32 (32P), radium-223, and (alpharadium) [29,30,31], as described in Table 2. The most widely used strontium isotope is an analog of calcium and selectively accumulates in metastatic lesions in the bones [32,33]. The therapeutic action is based on the emission of beta radiation, which destroys cancer cells with minimal damage to the surrounding healthy tissues. The therapeutic radioisotopes are usually injected into the bloodstream, allowing them to circulate and accumulate selectively at cancer sites, especially bone metastases. The isotope of Strontium-89, by mimicking calcium, penetrates into the cancerous bone matrix and emits beta particles that destroy nearby cancer cells. The isotope of Samarium-153 is chelated to Ethylenediamine Tetramethylene Phosphonic Acid (EDTMP), which has a high affinity for bone, especially of high osteoblastic activity, and through beta emissions causes tumor cell death, thereby helping to relieve bone cancer pain. Radium-223 (Alpharadin) is also able to mimic calcium and under conditions of increased bone turnover, as occurs in prostate bone metastases and together with high-energy alpha particles, is able to cause double-stranded DNA breaks in cancer cells with minimal damage to surrounding tissue [28].

4. The most common indication for the use of radioactive strontium isotopes is multiple, painful bone metastases that cannot be irradiated with external sources (teletherapy) due to their extensive distribution. The effects of isotopic therapy last from 3 to 12 months, and the onset of action may not become apparent until several weeks after the treatment. Occasionally, before the therapeutic effect occurs, there may be a transient increase in pain symptoms, in which case the patient may require a temporary increase in doses of analgesics [34,35]. Isotope treatment can be used in combination with RT, provided there is an appropriate time interval after prior monitoring of blood morphological parameters.

In RT, the probability of destroying all cancer cells increases as the number of target cells decreases. However, in RP, fewer cells do not result in a higher tumor control probability because the radiation is not uniformly delivered. If the radiation comes from a radionuclide on the tumor cell surface, fewer cells mean less energy is deposited into the targeted cells. This is partly offset by higher concentrations in smaller cell clusters compared to larger tumors.

**Table 2:** Radionuclides used in cancer pain relief.

Radionuclide	Cancer	Indications	Pain Relief Effect	Pain-Free Period
**Strontium-89 Chloride**	Prostate [36,37] breast lung, head and neck, colorectal [38]	Bone pain	63–88%	6 weeks–6 months
**Samarium-153-EDTMP**	lung, prostate [38,39] breast, osteosarcoma [40]	Bone pain	62–78%	3–8 months
**Radium-223-Dichloride**	Prostate [41,42]	Castration resistant prostate bone pain	41–72%	Up to 16 weeks
**Rhenium-186-HEDP**	Prostate [43], breast [44]	Bone pain	38% and 82%	5–12 months
**Rhenium-188-HEDP**	Prostate [45,46]	progressive hormone-resistant prostate carcinoma and bone pain	64–76%	6 weeks

## 3. The Molecular Mechanism of Radiotherapy

The fundamental physical cellular process in RT is ionization, along with the phenomenon of water radiolysis and the formation of hydroxyl free radicals, which trigger a series of biological and chemical consequences in the irradiated cell. The cytotoxic, anti-inflammatory, and anti-edema effects of RT lead to a reduction in tumor size and swelling around the tumor, thereby alleviating pressure on nearby structures of the nervous system and decreasing cancer infiltration into affected tissue structures, which contributes to a reduction in the severity of cancer pain [47]. Radiotherapy also exerts a hemostatic effect by obliterating small venous and arterial vessels within the tumor. Moreover, RT can directly affect pain receptors (e.g., nerves), changing their sensitivity or reducing their activity [48]. 

**Figure 1:**
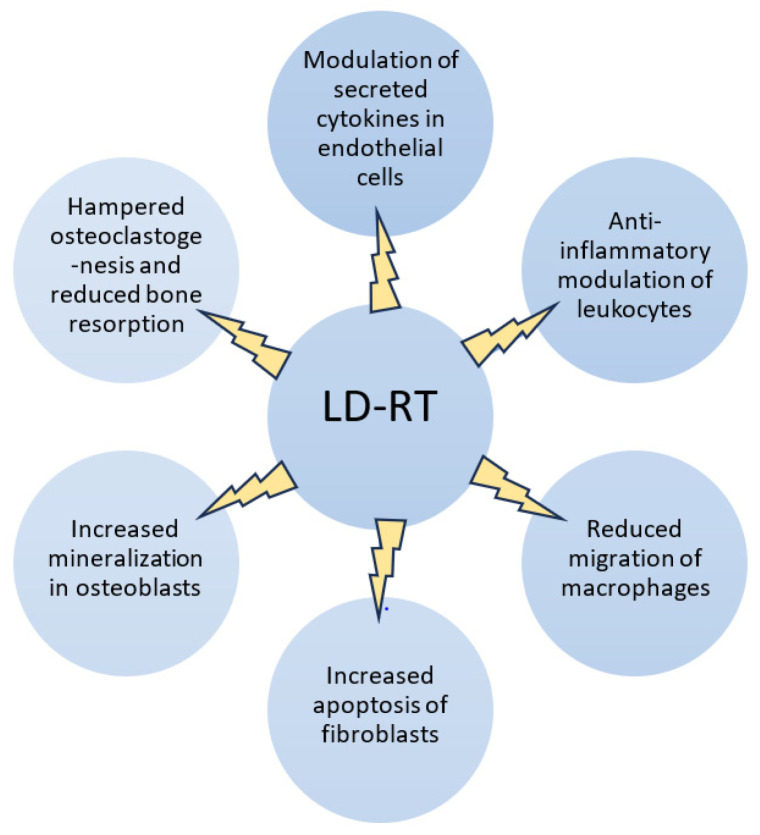
Various biological effects of low-dose radiotherapy (LDRT) on tissue in chronic inflammatory joint diseases.

The molecular effect of RT is a complex reaction which is supported by several mechanisms, as shown in Figure 1. First, by inducing apoptosis and necrosis in tumor cells, RT inhibits inflammatory mediators within the tumor and surrounding tissues. After sustaining damage, tumor cells decrease the release of cytokines such as TNF-alpha, IL-1, IL-6, and other mediators that sensitize pain receptors [49,50]. Second, ionizing radiation can directly affect nerve endings and alter their sensitivity by inducing changes in ion channel activity (such as the TRPV1 receptor) on sensory neurons, reducing their ability to transmit pain signals [51]. Third, RT modifies the tumor microenvironment by reducing tumor volume, which in turn decreases mechanical pressure on adjacent tissues, nerves, and blood vessels. Additionally, radiation-induced changes in blood flow and oxygenation in the tumor can result in reduced metabolic by-products that may irritate pain receptors [52]. Fourth, RT can activate the body’s natural pain-relieving mechanisms through the release of endorphins and other endogenous opioids in reaction to cellular stress and damage caused by radiation. Fifth, RT modulates the local tumor immune response, leading to anti-inflammatory effects. Immune cells that infiltrate the tumor in response to radiation can release factors that not only target cancer cells, but also reduce pain-inducing inflammation [53,54].

In addition, it has been demonstrated that RT reduces the activity of the cyclic adenosine monophosphate-protein kinase A (cAMP-PKA) signaling pathway, which is involved in both inflammatory pain and neuropathic pain [55,56]. The pro-inflammatory cytokines (such as TNF-*α* and IL-1*β* are activated and released from the astrocytes and microglial cells) are activators of the cAMP-PKA pathway in primary afferent neurons [57]. Preliminary results in rat models suggest that RT may suppress bone cancer pain through inhibition of abnormal activation of the cAMP-PKA signaling pathway, suggesting a new mechanism for the RT of bone cancer pain [58]. Finally, it is worth adding that RT affects the level of mineralization and bone density recalcification, which is associated with relief from bone cancer pain [59,60].

## 4. Radiotherapy as an Analgesic

Radiotherapy is a very common and effective treatment to alleviate cancer pain [61,62]. Pain relief after RT may be achieved in as many as 60–80% of patients [63,64]. The ionizing radiation can reduce the tumor or cancerous tissue infiltrates, which can ultimately contribute to a decrease in the intensity of perceived pain [65]. Radiotherapy is an effective pain management procedure. The most common clinical indications for the use of RT in alleviating cancer pain include: bone metastases, especially in the spine where there is a risk of spinal cord compression syndrome, brain metastases, painful cancer infiltrates in soft tissues, and cancerous obstruction of the pancreas and esophagus, which hinders painless swallowing [66,67]. Indications for RT also include superior vena cava syndrome and tumors blocking the airways, which make breathing difficult and may cause dyspnea, either with accompanying pain or occurring without pain [68,69].

### 4.1. Radiotherapy as a Mainstage Procedure in Painful Metastatic Bone Cancer

Bone metastases (BoM) are associated with very advanced cancer and occur in approximately 60% of cancer patients [70,71]. They most commonly affect patients with advanced breast, prostate, lung, kidney, or thyroid cancer [72,73]. The most serious complication of BoM is that of pathological fractures, which can lead to nerve damage, vascular injury, loss of joint stability, and even compression of nerves or the spinal cord in cases of metastatic lesions located in the spine.

Tests confirming the presence of BoM include X-rays, computed tomography, bone scintigraphy, magnetic resonance imaging, and PET scans, and other new molecular and hybrid imaging methods, including SPECT/CT, PET/CT, and whole-body MRI [74].

The most common sites for BoM include the thoracic spine, lumbar spine, sacrum, cervical spine, pelvis, ribs, and the humerus and femur [75]. Osteolytic BoM are associated with the destruction of trabecular and cortical bone due to the occlusion of medullary blood vessels by stimulated osteoclasts and are found in breast cancer, non-small cell lung cancer, thyroid cancer, and kidney cancer [76,77]. Osteoblastic BoM, which involves the overproduction of hard, brittle bone, typically occurs in small cell lung cancer, prostate cancer, or breast cancer.

The most frequent symptom in patients with BoM is pain, which is reported by more than two-thirds of patients. This pain is more frequently associated with metastases to the spine, pelvis, or ribs than with long bones [78]. It is often described as burning, stabbing, dull, nagging, or tearing. The pain intensifies during movement, physical activity, coughing, or sneezing. Notably, its intensity is lower or absent in the morning and increases throughout the day, peaking in the afternoon and evening, depending on the level of activity. Rest usually provides relief. In cases of BoM to the vertebral bodies, pain occurs while sitting or standing and subsides or decreases when lying down. However, if there is compression of the spinal cord, the pain sharply increases to severe levels when in a recumbent position, prompting patients to sleep in a semi-reclined or sitting position [79]. In patients with rib metastases, the pain worsens with deep breathing, and they tend to lie on the side affected by the metastases to immobilize the ribs during respiration.

Between 20% and 40% of cancer patients experience neuropathic pain, which is associated with higher pain intensity, poorer quality of life, and higher intake of analgesics [80,81]. Neuropathic mechanisms play an important role in the pathophysiology of cancer-induced bone pain and may cause metastatic bone pain refractory to standard pain treatments. Up to one-third of patients with bone metastases may suffer from neuropathic pain, which may be successfully treated with RT [82,83,84]. In many cases, patients with BoM live for many years, depending on the type of cancer, the number and volume of metastases, not only in the skeletal system but also in solid organs, the effectiveness of previous cancer treatments, the overall health of the patient, and the control of comorbidities [85].

One of the most effective methods for treating BoM is RT, which involves irradiating the selected metastatic focus. Radiotherapy is used in the following cases:

- Bone pain resulting from the presence of metastatic lesions, e.g., in the spine;
- Osteolytic metastases with significant bone loss that threaten fractures;
- Conditions following pathological bone fractures;
- Symptoms of spinal cord compression caused by tumor infiltration or a fragment of a fractured vertebra [86,87].

Radiation-induced pain relief in bone metastases is achieved by many mechanisms, which may include ossification, diminishing osteoclast activity in the bone microenvironment, and killing cancer cells along with a reduction in osteolysis. After RT, partial pain relief is achieved in 50–80% of patients, with complete relief observed in about 30% [59,88]. Typically, single, double, or five-fraction external beam irradiation is administered over one or two weeks. The effectiveness of these methods is comparable in terms of pain relief [89,90]. For the convenience of patients and their caregivers, single-dose radiotherapy is preferred for painful, uncomplicated BoM, although there is often a need for re-irradiation [91]. The decision regarding the individual treatment plan is made by the radiation oncologist, taking into account the type of cancer, the overall health of the patient, the predicted survival time, the presence of metastases in other locations, the feasibility of multiple visits to the radiotherapy center, and the patient’s preferences. Multi-fraction irradiation schemes (short course radiation therapy, for example, 10 × 3 Gy or 5 × 4 Gy) are usually proposed for patients in better overall condition with a longer expected survival, after orthopedic surgical procedures, and in cases of spinal cord compression syndrome [92] Patients with numerous painful BoM in worse overall health typically undergo single irradiation of the metastatic foci, but with a higher fractional dose (mostly 1 × 8 Gy [93]. Pain control persisting 3–6 months after RT is experienced by 35% of patients. In some patients, re-irradiation can be considered to prolong the pain relief effect after several weeks from the initial irradiation [92,93]. A limitation of the present review is that non-uniform primary end-points have been used in different studies (Table 3). Moreover, in the case of patients with neuropathic pain, multi-fraction irradiation schedules may be preferred to a single fraction, as this leads to longer durability of pain control with a higher remineralization rate of the irradiated vertebrae [94,95]. Furthermore, in patients with low-risk asymptomatic bone metastasis without gross osteolytic changes, especially female patients with pelvic, skull, and spine metastasis, there is an indication for RT in order to prevent the development of severe bone pain [96].

Stereotactic Body Radiation Therapy (SBRT) has emerged as a promising approach in the treatment of cancer, especially in managing BoM. While conventional RT remains ‘the gold standard’ in palliative care for pain management, the role of SBRT is becoming more distinct as it is increasingly recognized for its potential to improve prognosis, rather than simply alleviating pain. This distinction is crucial when considering the role of SBRT in clinical practice, particularly for patients with BoM.

One of the key benefits of conventional RT is its well-established ability to reduce pain associated with BoM. The radiation can shrink or slow the growth of metastatic tumors in bone, decreasing pressure on surrounding tissues and nerves, which provides meaningful relief for patients, allowing them to regain functionality and improve their quality of life. Furthermore, the procedure is non-invasive, and the side effects are generally manageable. SBRT, on the other hand, represents a more advanced and precise approach. Unlike conventional RT, which typically involves fractionated doses spread over time, SBRT delivers high doses of radiation in fewer (1–5) sessions. The higher RT dose can be more effective in controlling tumor growth, especially in difficult-to-reach or previously treated areas. The radiation beams are precisely targeted at the tumor using advanced IGRT, allowing for a highly concentrated dose to be delivered directly to the metastatic site with minimal impact on surrounding healthy tissues.

SBRT for BoM is increasingly viewed not as a purely palliative measure, but as a treatment aimed at improving prognosis [97]. In clinical practice, SBRT is often associated with better tumor control, which can result in prolonged survival and improved outcomes in patients with oligometastatic disease or those who are otherwise well enough to tolerate more aggressive treatment. Several studies have shown that SBRT for BoM is superior to EBRT in terms of the 3-month pain response rate and with regard to a higher complete response rate and long-term pain control [98,99]. However, SBRT is not primarily considered a palliative treatment for pain in the same way as conventional RT. Although pain relief can occur as a secondary benefit of SBRT, the primary goal of the therapy is often to provide long-term control of metastatic lesions, potentially improving overall survival rates. The high doses of radiation delivered via SBRT can halt or even shrink tumors in the bone, leading to improved structural stability and fewer complications from the metastatic disease. This, in turn, can contribute to better functional outcomes and a reduction in the need for other interventions, such as surgery or long-term use of analgesics [100,101]. The key distinction lies in the intent and application of the treatment: conventional RT is geared toward immediate, short-term symptom relief, while SBRT is increasingly focused on longer-term disease control, which may indirectly result in pain relief over time as tumor size reduces or stabilizes. This approach is particularly beneficial for patients with low metastatic burden who are candidates for aggressive treatment [102,103].

Both SBRT and conventional RT play vital roles in the management of cancer, particularly in patients with BoM. Conventional RT remains a cornerstone of palliative care for pain relief, offering symptomatic improvement for many patients in need of immediate care. SBRT; however, has emerged as a high-precision treatment that, while potentially offering pain relief, is primarily aimed at improving prognosis and controlling the progression of metastatic disease. As such, SBRT should not be viewed as a first-line palliative treatment for pain, but rather as an option for selected oligometastatic cancer patients.

In summary, the decision on RT fractionation should be supported by guidelines designated by the American Society for Radiation Oncology (ASTRO) Evidence-Based Guidelines or the European Society for Radiation Oncology (ESTRO) [104,105,106]. According to these recommendations, it is considered that pain relief is equivalent after a single 8 Gy fraction, 20 Gy in 5 fractions, 24 Gy in 6 fractions, and 30 Gy in 10 fractions for patients with previously unirradiated painful bone metastases. A single fraction treatment in particular may be considered for patients with poor overall prognosis. Re-irradiation in the case of symptomatic peripheral bone metastases or in spine lesions should be used while adhering to normal tissue dosing constraints.

**Table 3:** Clinical studies demonstrating the analgesic effect of irradiation in painful bone metastases depending on various dose and radiotherapy fractionation.

Study	RT Scheme	Complete Pain Response	Partial Pain Response
Steenland et al., 1999 [107]	1 × 8 Gy	72%	37%
	6 × 4 Gy	69%	33%
Koswing et al., 1999 [108]	1 × 8 Gy	79%	31%
	10 × 3 Gy	82%	33%
Roos et al., 2005 [94]	1 × 8 Gy	61%	15%
	10 × 3 Gy	53%	18%
Hartsell et al., 2005 [109]	1 × 8 Gy	65%	15%
	10 × 3 Gy	66%	18%
Foro Arnalot et al., 2008 [89]	1 × 8 Gy	75%	15%
	10 × 3 Gy	86%	13%
Nongkynrih et al., 2018 [109]	1 × 8 Gy	80%	20%
	5 × 4 Gy	75%	20%
	10 × 3 Gy	85%	20%
Nguygen et al., 2019 [110]	1 × 12 Gy–16 Gy	55%	52%
	10 × 3 Gy	34%	19%
Nguygen et al., 2023 [111]	2 × 12 Gy	83–94%	-
	
Ryu et al., 2023 [112]	1 × 16–18 Gy	60%	-
	1 × 8 Gy	41%	

For patients with widespread involvement of the skeletal system, half-body irradiation may be considered, which typically alleviates pain in about 70% of patients for 2–3 months [113]. The upper, middle, or lower half of the skeleton may be irradiated [114,115]. In some patients with stable, oligometastatic (up to five metastatic foci) cancer, stereotactic irradiation of single BoM, such as in the spine, may be considered [116,117]. The stereotactic radiotherapy technique, which delivers a high ablative biological dose in one to several fractions in a short treatment course, involves administering one, two or three high fractional doses of ionizing radiation to a precisely defined metastatic focus, guided by imaging studies with careful planning of the irradiated area based on magnetic resonance imaging (MRI) or positron emission tomography (PET). Recent studies show that the effects of this treatment last longer and usually do not require re-irradiation [117,118].

In patients with multiple BoM, where the use of teletherapy is challenging, isotopic therapy should be considered. In this case, particular indications pertain to metastatic foci in long bones (femur, tibia, bones of the hand) or in the spine and pelvis, which are most prone to pathological fractures. Isotopic treatment is indicated even with mild pain symptoms, as it reduces pain and strengthens the bones at the site of the metastases.

### 4.2. Radiotherapy as an Effective Analgesic Procedure in Advanced Head and Neck Cancer Patients

Patients with advanced head and neck cancer often suffer severe pain as a result of extensive soft tissue tumor infiltration, which is often difficult to alleviate pharmacologically. These patients frequently take high doses of strong analgesics without achieving satisfactory pain relief. Radiotherapy is used in the form of short-course treatment both in order to treat extensive cancer infiltration and to relieve pain [119,120]. This RT procedure not only provides effective relief from cancer pain after just a few days, but may also reduce the need for pain medication, especially strong opioids [121].

### 4.3. Radiotherapy as an Effective Procedure in Inflammatory Joint Diseases

Cancers occur most often in older people with comorbidities, especially degenerative joint diseases such as osteoarthritis. However, RT for degenerative joint disease is not a first-line treatment and it is generally used when other options fail. It is important to note that RT in most cases is not a cure for the underlying degenerative condition but may help in managing symptoms. An interesting and important feature of RT with low-dose ionizing energy (single dose of 0.5 to 1 Gy and a total dose of 3 to 6 Gy) which has been recognized since the early 20th century is its analgesic effect in treating inflammatory and degenerative musculoskeletal diseases (MSDs) such as plantar fasciitis and epicondylitis humeri. The exact mechanism remains unclear, but it is believed that low-dose radiotherapy triggers a complex anti-inflammatory response, which is shown in Figure 1. The effects of anti-inflammatory radiation involve functional modulation of the adhesion of white blood cells to activated endothelial cells and regulation of the induction of nitric oxide synthase in activated macrophages [122,123]. This process most likely involves inhibiting leukocyte accumulation, transfer, and differentiation into macrophages, while also reducing the production of pro-inflammatory cytokines and nitric oxide, which regulate vascular permeability and help suppress the inflammatory cycle [124,125].

Moreover, it has been noticed that after low-dose RT, pro-inflammatory cytokines were generally slightly reduced, but IL-6 is significantly increased. IL-6 is a pro-inflammatory cytokine involved in various pathways, including bone metabolism. While it typically promotes bone resorption and inflammation, it can also have anti-resorptive effects by increasing osteoprotegerin, which inhibits osteoclastogenesis [126]. To sum up, RT is more effective in healing superficial soft tissue diseases or inhibiting cartilage degeneration in osteoarthritis, where the therapeutic effect in the case of osteoarthritic lesions reaches up to 75% [127].

### 4.4. Factors Predicting RT Effectiveness

It is likely that the future direction of research in helping to alleviate cancer pain will be to identify predictors or markers for response to palliative RT in order to classify patients according to successful responses to RT. It is well known that performance status is one of the most important variables in predicting RT response. Moreover, breast or prostate cancer patients also have a significantly better response after analgesic RT [128]. Furthermore, the presence of soft tissue expansion outside bone has been found to predict better RT response in patients with painful bone metastases. It has been shown that inflammation measured with CRP was not a predictor for RT response, but patients using corticosteroids had significantly lower response rates with painful bone metastases [129,130]. The latest research emphasizes that educational and organizational strategies are needed to reduce the proportion of patients with inadequate pain management. It is well known that the earlier RT is used in painful bone metastases, the higher its effectiveness [131]. Referring patients for palliative RT earlier may improve the probability of better pain responses and treatment outcomes. Finally, it is worth adding that the effectiveness of RT can be greatly improved by using new technologies to safely deliver higher doses of radiation while sparing healthy tissues as much as possible.

## 5. The Other Side of the Coin in the Effect of Radiotherapy

### 5.1. Pain as a Consequence of Radiotherapy

Radiotherapy not only kills cancer cells but may also affect adjacent non-cancerous cells by triggering the release of various cytokines and inflammatory mediators from damaged cancer cells. The inflammatory reaction can cause fibrosis, atrophy, and ulceration of the tissues, including vessels and nerves, and consequently, nerve damage may result in increased pain [132]. In rare cases, radiation-induced neuropathy may occur. The exact mechanism of this painful condition is still unclear. Radiation-induced neuropathic pain is often progressive, unresponsive to conservative treatment, and at times irreversible. The main mechanism of radiation-induced neuropathic pain, such as radiation-induced brachial plexopathy after radiotherapy for breast carcinoma, is perineural fibrosis and ischemia, subsequently leading to myelin destruction and axonal injury [133].

**Figure 2:**
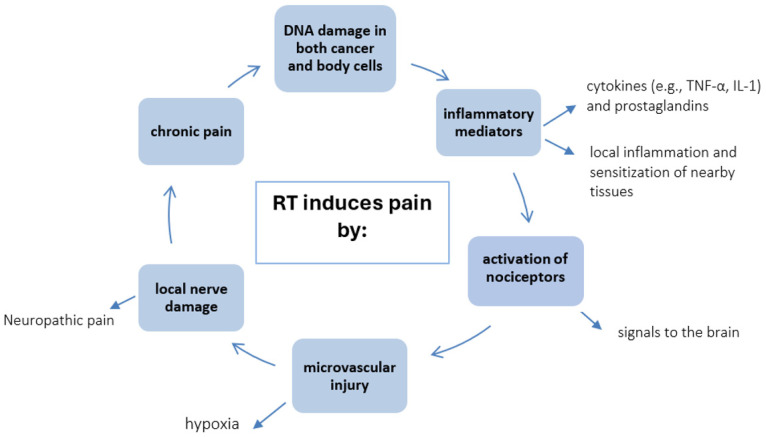
The patho-mechanisms of pain induced by radiotherapy (RT).

Radiotherapy may produce various types of post-RT chronic pain syndromes, including peripheral nerve entrapment, radiculopathy, myelopathy, noncardiac chest pain, pelvic pain, osteonecrosis, and other soft-tissue damage after exposure to RT [134]. The neuropathic pain may be successfully treated with oral pharmacotherapy, systemic drug infusion with lidocaine, or with spinal cord stimulation [135,136], but radiation-induced neuropathic pain is often unresponsive to conservative treatment. Systemic lidocaine treatment is worth applying in patients with intractable radiation-induced neuropathic pain, even if it is a centrally sensitized state, as a rescue-treatment modality. The main mechanism of action of systemically administered lidocaine is blocking peripheral and central voltage-gated sodium channels, and increasing spinal inhibitory glycinergic neurotransmission mediated by *N*-methyl-d-aspartate receptors. Systemic lidocaine treatment may reduce opioid consumption and relieve pain in patients with intractable radiation-induced neuropathic pain [137,138]. The various pain mechanisms occurring after RT are presented in Figure 2.

### 5.2. Painful Complications After RT

The most common side effects of RT to the spine may include temporary worsening of back pain, sore throat, heartburn, and nausea, depending on the level of the spine being treated. After RT, patients may experience temporary fatigue, and because ionizing radiation penetrates through the skin, it may cause the treated area to look and feel sunburned—a condition called radiation dermatitis [139,140]. Most of these are transient early reactions and do not require treatment. However, RT may also cause late consequences. Radiation fibrosis syndrome is a general term describing a myriad of clinical conditions associated with RT, typically caused by an increased accumulation of thrombin in tissue, which may lead to progressive fibrotic tissue sclerosis. Radiation fibrosis syndrome may present during RT or years after treatment and can affect any tissue type, including skin, muscles, tendons, viscera, and nerves. Its effects can have significant functional implications, such as pain, loss of sensation, and weakness [141]. A comparatively rare phenomenon is radiation mononeuritis described as radiation-induced injury at the nerve root or plexus [142]. Late mononeuropathy is a relatively rare complication, but should be considered in patients presenting with neuropathy after RT [143]. Other complications following RT which cause pain include osteoradionecrosis (ORN) in head and neck cancer patients, and soft tissue necrosis, specifically manifesting as ulceration and cavitation [144]. Erly ORN is related to higher radiation doses (>70 Gy), late ORN is multifactorial, involving radiation-related cellular injury and impaired osteoblast function, as well as vascular damage resulting in a chronically hypoxic microenvironment [145,146].

Radiation can also cause damage to the bones of the spine, which may result in a reduction in the patient’s height or change in the shape (curvature) of the spine [147]. In a study with rats, it was observed that two weeks after irradiation the three-dimensional bone structure model became coarse, and the trabecular structure continued to thin and disrupt after irradiation, while significant changes in the bone mineral density were not noticed [148]. In vitro studies show that high-dose radiotherapy (RT) significantly damages the viability and function of bone cells, including osteoclasts, osteoblasts, and osteocytes. In vivo animal models reveal that high-dose RT causes morphological changes, inhibits bone repair, and increases bone fragility. Clinical data indicate a rising risk of bone damage, such as fractures over time following high-dose RT [149,150]. Most importantly, osteopenia and osteoporosis increase the risk to bones after RT and are significant problems among cancer patients. Numerous clinical studies have shown that osteoporosis is also a risk factor for developing sacral insufficiency fractures and vertebral body fractures after RT [151,152]. Table 4 summarizes these two sides of RT as a double-edged sword in association with cancer pain.

### 5.3. Pain Flares as a Temporary Side Effect

A flare-up of pain is a common side effect of radiopharmaceutical and hormonal therapies, with incidence rates for external beam RT ranging from 2% to 44% [170,171]. Hird et al. reported a 40% incidence of pain flares, mostly occurring within the first 5 days of treatment [172]. These flares may be linked to the rapid release of inflammatory cytokines, and dexamethasone, due to its anti-inflammatory effects, may help to reduce or prevent them [173]. How et al. found that dexamethasone, compared to placebo, reduced pain flare incidence, nausea, and improved functional activity and appetite without serious side effects [174].

### 5.4. Promising Technological Progress in RT

The future of RT in alleviating cancer pain holds significant promise, driven by ongoing advancements in technology in treatment planning. To continue improving the effectiveness of RT, several strategies should be explored and implemented. A significant focus of future developments in RT lies in increasing the precision of treatment delivery. With the increasing availability of varying genetic and molecular profiles of cancer tumors, RT should become more personalized to cater to the unique characteristics of the individual patient’s cancer. One promising approach is adaptive RT, where the treatment plan can be modified in real time based on changes in the tumor size, shape, and position. This approach will become more efficient with advanced imaging techniques like MRI and PET scanning, helping adjust radiation doses to maximize effectiveness and minimize damage to healthy tissue.

Incorporating Artificial Intelligence (AI) and Machine Learning (ML) to revolutionize RT treatment planning and delivery will make the process faster, more accurate, and more efficient. These technologies can assist in the process of automated tumor and organs at risk contouring to improve consistency in treatment processes [175,176]. Moreover, AI-powered systems can optimize RT dose distributions, allowing for more customized and precise delivery of RT, which in turn, should help to reduce side effects and enhance pain relief.

Predicting Outcomes algorithms based on an analysis of large amounts of patient data can help to establish which treatment regimens are most likely to relieve pain and shrink tumors, allowing for more informed decisions about treatments, including RT [176]. Tailoring combinations of RT with chemotherapy, immunotherapy, or targeted therapies based on the individual’s genetic profile will allow for more effective cancer treatments, reducing the likelihood of recurrence and improving the overall quality of life by alleviating pain and improving long-term survival. Ultimately, the future of RT is promising due to dynamic technological progress in this area, which bodes well for providing more effective treatments that increase survival rates and lead to improvements in long-term remission.

## 6. Conclusions

Radiation therapy remains a key cancer treatment, especially for cancer pain. As clinical outcomes improve, minimizing radiation-related toxicities has become a priority. Radiotherapy works in conjunction with pharmacological, radioisotope, or molecular therapy, thereby increasing the analgesic effect to enhance therapeutic efficacy, not only in cancer, but also in chronic inflammatory joint diseases. Moreover, RT, despite its documented analgesic effects, may also induce or intensify pain in certain clinical situations, as described above. Most of these side effects generally dissipate over time. To sum up, RT, in addition to its analgesic effects, can cause serious, undesirable side effects in terms of pain induction, which can make it something of a double-edged sword in cancer treatment. Many of these side effects present difficult challenges to patients, and their recognition and treatment can significantly improve patients’ health, long-term survival, and QoL.

## Figures and Tables

**Table 1:** Types of brachytherapy are categorized according to the methods used for placement of the applicator.

Type of Brachytherapy	Location of Applicator
interstitial	The applicator is placed inside the tumor, e.g., prostate cancer [9]
surface	A contact applicator is used in the treatment of skin cancers [10]
intracavitary	The radiation source is placed within body cavities, e.g., in the uterus, oral cavity cancers, or spinal canal cancers [11]
intraluminal	An applicator is inserted into the lumen of a cancer-infiltrated bronchus, e.g., irradiation of an intrabronchial lesion leads to its reduction and improved bronchial patency, thereby decreasing dyspnea and cancer pain [12]
intraoperative	An applicator is placed in the post-operative cavity, e.g., following removal of a breast tumor [13]

**Table 4:** Radiotherapy (RT) is both an analgesic and a pain factor in various clinical situations.

Radiotherapy as a Double-Edged Sword—Examples	
Analgesic in:	Pain Factor:
○ painful cancer infiltrates in head and neck [153,154]	skin irritation with redness, dryness, itching after skin cancers RT [155]
○ painful vena cava superior syndrome [156]	mucosal pain as a result of early post-radiation reaction in head and neck or esophagus cancers [157,158]
○ painful bone metastases	neuropathic chest wall pain following RT for lung or breast cancers [159,160]
○ neuropathic pain of pancreatic cancer [161,162]	bowel symptoms after pelvic RT [163]
○ pelvic pain in advanced colon cancer [164]	headaches after brain tumors RT [165]
○ spinal cord compression [166,167]	perineal pain after gynecological tumors RT [168,169]
